# 

**Published:** 1895-12-07

**Authors:** 


**Notices of Births, Deaths, and Marriages** are inserted in this column at a charge of 2s. 6d. for an announcement not exceeding four lines.

**Notice to Advertisers and Subscribers.**

All Advertisements, Orders for Copies of The Hospital and Business Communications should be addressed to **THE MANAGER, (Not to The Editor),** The Hospital, 428, Strand, London, W.C.

The Cost of Subscription, post free, is as follows:—Per week, 2½d.; per quarter, 2s. 8d.; per half-year, 5s. 3d.; per annum, 10s. 6d. Foreign:—Per Annum, 15s. 2d.; payable, in all cases, in advance.

### NOTICE TO CORRESPONDENTS.

All MS., Letters, Books for Review, and other matters intended for the Editor should be addressed The Editor, The Lodge, Porchester Square, London, W.

The Editor cannot undertake to return rejected MS., even when accompanied by stamped directed envelope.

**"Burdett's Hospital Annual," 1895.**

Sixth year of publication. 950 pp. Scarlet cloth, gilt lettered. Price 5s. A most complete guide to British, Colonial, Indian, and American Hospitals, Dispensaries, Nursing and Convalescent Institutions, and Asylums. A few complete sets of the preceding five issues of the "Annual" may still be obtained on application to the Publishers, The Scientific Press (Limited), 428, Strand, London, W.C.

**NOTICE.—Vol. XVIII. of "The Hospital" (April to September, 1895), in handsome cloth binding, is now on sale at the Office of this Journal. Price 7s. 6d. Cases for binding the Numbers contained in this Volume, 1s. 6d. Index to Vol. XVIII., 2½d., post free.**

**The Monthly Part for December is now ready, price 6d., by post, 8d.**

### SCRAPS AND GLEANINGS.

A woman's medical college has just been established at Kansas City.

Mr. David Stewart, of Banchory, has presented £210 to the Aberdeen Royal Infirmary Jubilee Extension Fund.

The Paris Académie de Médecine has instructed a commission to investigate the best methods of combating malaria.

A legacy of £200 has been received by the treasurer of St. Helens Cottage Hospital, Manchester, from the late Mrs. Earlam.

There were no less than 1,168 persons practising medicine in Bavaria, without a legal qualification, in 1894. Of this number 865 were men, and 302 were women.

A legacy of £100 has been received from Mr. John Croombie by the treasurer of the Convalescent Hospital in connection with the Aberdeen Royal Infirmary.

The first house-to-house collection on behalf of the funds of the Monkwearmouth and Southwick Hospital was made last week, the proceeds being devoted to the extension fund.

Messrs. Humphreys (Limited), of Knightsbridge, the well-known makers of iron hospitals, are sending hospitals out to the goldfields of South Africa this week and also to Spain, for 50 beds.

A cheque for £1,000 has been handed to the treasurer of the Royal Portsmouth, Portsea, and Gosport Hospital for the use of the institution, the sole condition being that the identity of the donor is not disclosed.

His Royal Highness the Duke of Cambridge, not the Duke of Richmond, as erroneously stated, presided at the dinner in aid of the Building Fund of the Royal Hospital, Richmond, at the Star and Garter Hotel.

The Paris Académie de Médecine has been approached as to the establishment of an anti-corset measure. To bring this more readily about, the enthusiastic reformer suggests the levelling of a tax on all corsets.

An institution, after the fashion of the Nazareth House, Hammersmith, is planned to be erected at Johannesburg. Mr. Alfred Beit, the South African millionaire, has laid the foundation-stone of this building.

The Metropolitan Gospel Mission Seaside Convalescent Cottage Association have acquired the freehold of four of the cottages at Bognor which the society have rented for their charitable purposes for fifteen years.

The Redruth St. John's Ambulance Brigade has been presented with a solid leather case containing all the appliances necessary in rendering first aid to the wounded. The donor of this useful gift is Mr. Caspar R. Laurie, L.R.C.P.

The Duchess of York has consented to become president of the endowment fund in connection with the Ladies' Association of the Great Northern Central Hospital. Towards this fund Her Royal Highness has promised a donation of £20.

The secretary of the Charing Cross Hospital writes that a well-dressed woman last week took upon herself to collect money for a fund for purchasing blankets for the hospital. There is no such fund in existence, and the collection is quite unauthorised.

The Associated Societies for the Protection of Women and Children has been doing a good work for over 40 years. Although there is not any striking feature in this year's report, yet the society maintains its character as a beneficient agency.

### The Student of Medicine.

### APPOINTMENTS.

F. T. Auden, M.B., C.M.Edin., appointed District Surgeon for the Rustenburg District of the South African Republic. F. H. Carlyon, M.B., C.M.Edin., appointed Honorary Surgeon to the Royal Cornwall Infirmary; also appointed Divisional Surgeon to the Cornwall County Constabulary. W. Easby, M.D.Brux., L.R.C.P., L.R.C.S.Edin., appointed Medical Officer of Health to the Peterborough Rural District. Dr. Ewing, appointed Medical Officer to the East and West Ardley District Council. G. W. S. Farmer, M.A., M.B., M.Ch.Oxon., F.R.C.S, Radcliffe Travelling Fellow, appointed Examiner in Human Anatomy at the University of Oxford. F. W. Hall, M.D. and M.S.Lond., M.R.C.S.Eng., L.R.C.P.Lond., appointed Honorary Assistant Physician to the Sydney Hospital, Sydney, N.S.W. C. M. Hardy, M.B., B.S.Durh., appointed Medical Officer of Health to the Croft Rural District Council. Mr. Hasluck, appointed Deputy Medical Officer of the Kidderminster Union Workhouse. G. E. Manning, M.R.C.S.Eng., L.R.C.P.Lond., appointed Resident House Physician to the Sunderland Infirmary. W. Ranson, L.R.C.P., L.R.C.S.Edin., appointed Registrar and Assistant to the Surgeon at the County Down Infirmary. J. A. Robertson, M.D.Glasg., M.B., C.M., appointed Medical Officer of Health to the Stilton Rural District. H. C. Strover, L.S.A., L.A.H. Dub., appointed Medical Officer for the Tempsford District of the Biggleswade Union. W. M. Willis, M.R.C.S.Eng., L.R.C.P. Lond., appointed House Surgeon to the Bristol Hospital for Sick Women and Children.

### VACANCIES.

The following vacancies are announced this week, the amount of salary in each case being given after the name of the institution:—

### Resident Medical Officers.

Central London Ophthalmic Hospital, 238A, Gray's Inn Road, W.C.—House Surgeon. Rooms, coals, and light. Applications to the Secretary by December 10th.

City of London Hospital for Diseases of the Chest, Victoria Park, E.—House Physician. Appointment for six months. Board and residence provided, and salary at the rate of £30 per annum. Applications to the Secretary by December 12th.

Ennis District Lunatic Asylum.—Assistant Medical Officer, doubly qualified, unmarried, and not more than 30 years of age. Salary £100 per annum, with furnished apartments, rations, washing, fuel, light, and attendance. Applications to Dr. Gelston, Resident Medical Superintendent, by December 13th.

Glasgow Eye Infirmary.—Resident Assistant House Surgeon. Salary £50 per annum, with apartments and board. Applications to William George Black, Secretary, 88, West Regent Street, Glasgow, by December 10th.

North-Eastern Hospital for Children, Hackney Road, Shoreditch, N.E.—House Physician; doubly qualified. Appointment for six months; at the expiration of this term he will be required, if eligible, to serve as House Surgeon for a further period of six months. Salary as House Physician at the rate of £60, and as House Surgeon at the rate of £80 per annum. Junior House Physician for six months; doubly qualified. No salary, but board and lodging, including washing, provided. Applications to the Secretary, 27, Clement's Lane, E.C., by December 9th.

Norfolk and Norwich Hospital.—House Physician and House Surgeon; doubly qualified; unmarried, and under 30 years of age. Salary for each office £60 per annum, with board, lodging, and washing. Applications to Poole Gabbett, Secretary, by December 10th.

Royal Berks Hospital, Reading.—House Surgeon and House Physician. Salary in each case £60 per annum, with board, lodging, and washing. Also Assistant Medical Officer, with board, lodging, and washing provided, but not salary. Appointments for six months. Applications to the Secretary before December 9th.

St. Mark's Hospital, City Road, E.C.—House Surgeon; must possess a surgical qualification. Salary £50 per annum, with board and lodging. Applications to the Secretary by December 9th.

Swansea General Hospital.—House Surgeon. Salary £50 per annum, with board, residence, washing, and attendance. Applications to Jno. W. Morris, Secretary, 9, Castle Street, Swansea, by December 16th.

Warneford Hospital, Leamington.—House Surgeon. Salary £100, with board, lodging, and washing. Appointment for six months, subject to re-election. Applications to the Secretary before December 14th.

West London Hospital, Hammersmith Road, W.—House Physician and House Surgeon. Appointments for six months. Board and lodging provided. Applications to R. J. Gilbert, Secretary-Superintendent, by December 18th.

York Lunatic Asylum, Bootham, York.—Assistant Resident Medical Officer. Salary £100 per annum, with board, washing, and attendance. Applications, addressed to the Committee, to be sent under cover to R. D. Horne, Secretary, by December 11th.

### Non-Resident Medical Officers.

North-Eastern Hospital for Children, Hackney Road, Shoreditch.—Ophthalmic Surgeon; must possess surgical qualification. Applications to the Secretary, 27, Clement's Lane, E.C., by December 9th.

### Medical Appointment.

YORK LUNATIC ASYLUM.

The Scientific Press (Limited), 428, Strand, London, W.C.

*In the Press. Price 5s.*

### BURDETT'S OFFICIAL NURSING DIRECTORY

A DIRECTORY, NOT A REGISTER.

**The above work may be subscribed for at a reduced price by those whose names appear in the book, and who supply full particulars on a form which can be obtained upon application to the Publishers,**

THE SCIENTIFIC PRESS (Lim.), 428, Strand, London, W.C.

### IMPORTANT NOTICE.

OUR ISSUE OF DECEMBER 14TH WILL CONSIST OF A

### SPECIAL DOUBLE CHRISTMAS NUMBER,

With which will be presented a handsome presentation plate of

**H.R.H. THE PRINCE OF WALES.**

Taken from an entirely new and hitherto unpublished photograph.

This plate will be a companion plate to that of H.R.H. the Princess of Wales, given with our Christmas Number in December last.

**2d.** PRICE AS USUAL. **2d.**

In order to secure copies orders should be sent in at once to

THE MANAGER, "THE HOSPITAL," 428, STRAND, LONDON, W.C.

**NURSING BOOKS AT FULL DISCOUNT.**

*LIST POST FREE.*

The Theory and Practice of Nursing (Percy Lewis, M.D.), 3/6 for **2/8.**
The Nurse's Dictionary (Honnor Morten), 2/- for **1/6.**
The Nurse's Case Book, 6d. for **3½d.**

### STANDARD TEXT-BOOKS ON NURSING, &c.

*Now Ready, 8th Edition. Greatly enlarged and thoroughly revised. Crown 8vo., cloth gilt, 3s. 6d.*

### NURSING: ITS THEORY AND PRACTICE,

BEING A

### COMPLETE TEXT-BOOK OF MEDICAL, SURGICAL, AND MONTHLY NURSING.

**By PERCY G. LEWIS, M.D., M.R.C.S., L.S.A., &c., &c.**

To this edition have been added three chapters on Monthly Nursing, together with additions to the chapters on Blood Diseases, on Antiseptics, on the Nursing of Children, and on Private Nursing. Under these headings will be found a short account of the new Aseptic Treatment, the Antitoxin and Serum Treatments, and the Treatment by Thyroid Extracts.

These additions, combined with the revision throughout of the text, render this work the best and most complete Text-book on Nursing in the market, and strengthen its position as a Classic in the Training Schools of the World.

Now Ready. With numerous Illustrations, demy 8vo., blue buckram, gilt, 3s. 6d.

### DIET IN SICKNESS AND IN HEALTH.

**By Mrs. ERNEST HART,**

*Formerly Student of the Faculty of Medicine of Paris, and of the London School of Medicine for Women.*

With an Introduction by Sir **HENRY THOMPSON, F.R.C.S., M.B. Lond.**

"Does not ask the reader blandly to accept such and such a diet for such and such complaint, but explains to him, briefly and simply, the why and wherefore, and gives in each case excellent physiological lessons, illustrated with comprehensive diagrams. Another good point is its cheerful sensible tone. The book is excellent in every way, and may be warmly recommended to the enormous number of people who, for one reason or another, have to be careful about their diet."—*Westminster Gazette.*

"Mrs. Hart not only expounds the principles of dietetics with insight and authority, applying them particularly and generally to all forms of disease and disability, but she is also able to act as a guide to the actual practice of dietetics. A really useful handbook to all invalids, nurses, medical students, and their kind."—*Science Siftings.*

Second and Revised Edition. Demy 16mo., suitable for the apron pocket, handsomely bound in cloth, 140 pp. Price 2s.; in handsome leather, gilt, price 2s. 6d. net.

**THE NURSES' DICTIONARY OF MEDICAL TERMS AND NURSING TREATMENT.**—Compiled for the use of Nurses by HONNOR MORTEN. Containing descriptions of the principal Medical and Nursing Terms and Abbreviations, Instruments, Drugs, Diseases, Accidents, Treatments, Physiological Names, Operations, Foods, Appliances, &c., &c., encountered in the Ward or Sick-room.

200 pp., Crown 8vo., cloth, 3s. 6d. Profusely Illustrated with Original Drawings, with list of Instruments required, and a Glossary of Terms.

**OPHTHALMIC NURSING.** By SYDNEY STEPHENSON, M.B., F.R.C.S.E., Surgeon to the Ophthalmic School, Hanwell, W., &c., &c. This is a valuable contribution to Nursing literature on a subject hitherto never handled with special reference to Nursing. On account of its compact and clear arrangement, and its numerous illustrations, it is specially recommended to the use of the Nursing Profession.

Second Edition, with a New Chapter and several Coloured Plates Illustrative of Feeding Bottles used in ancient times, &c. Crown 8vo., imitation cloth, 5s. With 10 plates and many Illustrations in the Text, and Fac-simile Autograph Letters from the late Sir Andrew Clark, M. Pasteur, &c.

**INFANT FEEDING BY ARTIFICIAL MEANS:** A Scientific and Practical Treatise on the Dietetics of Infancy. By S. H. SADLER, Author of "Suggestions to Mothers," "Management of Children," "Education."

Crown 8vo., cloth gilt, Illustrated, price 5s.

**HELPS IN SICKNESS AND TO HEALTH:** Where to Go and What to Do. Being a Guide to Home Nursing and a Handbook to Health in the Habitation, the Nursery, the Schoolroom, and the Person, with a chapter on Pleasure and Health Resorts. By HENRY C. BURDETT, Author of "Hospitals and Asylums of the World," "Pay Hospitals of the World," "Cottage Hospitals, General, Fever, and Convalescent," &c., &c.

"It would be difficult to find one which should be more welcome in a household than this unpretending but most useful book."—*The Times.*

Important New Handbook of Midwifery. Handsomely bound in leather, 260 pp., profusely illustrated, price 6s.

**A PRACTICAL HAND-BOOK OF MIDWIFERY.** By FRANCIS W. N. HAULTAIN, M.D. A Practical Manual produced in a portable and convenient form for reference.

Third Edition, enlarged and partly re-written, crown 8vo., cloth gilt, about 200 pp., price 2s. 6d.

**HOSPITAL SISTERS AND THEIR DUTIES.** By EVA C. E. LUCKES, Matron to the London Hospital. The favourable reception accorded to the first two editions of this useful and interesting work, and the regular demand for copies of it, encourage the publishers to place this third edition, greatly improved and enlarged, before the Institutions. The experience and position of its author entitle it to authority, and every Matron, Sister, or Nurse who aspires to become a Sister should read the advice and information contained in its pages.

Crown 8vo., 500 pp., Second Edition, 7 Plates, 18 Illustrations, Charts, &c., price 7s. 6d. net.

**NURSING.** By ISABEL ADAMS HAMPTON.

"It is not too much to say that in no single volume yet published has the subject been so scientifically, carefully, and completely treated as it has been by Miss Hampton. . . . Whether as a teaching manual or as a book of reference, the thoroughly practical instruction which it conveys is sure to obtain a wide circulation."—*The Hospital.*

Crown 8vo., cloth gilt, containing many Plates and Illustrations, price 2s. 6d.

**SPINAL CURVATURE AND AWKWARD DEPORTMENT:** Their Causes and Prevention in Children. Dr. GEORGE MULLER, Professor of Medicine and Orthopædics, Berlin. English Edition, edited and adapted by RICHARD GREENE, F.R.C.P.

"An able essay upon the effect produced by exercise, attitude, and movement upon the growth and development of the body. He further gives directions which any intelligent parent or teacher could carry out with the help of very simple apparatus, showing how to avoid or, in early cases, to cure certain malformations which, in the majority of cases, arise either from the neglect of very simple hygienic rules or from carelessness."—*Daily Chronicle.*

LONDON: THE SCIENTIFIC PRESS (LIMITED), 428, STRAND, W.C.

### PAMPHLETS ON NURSING.

No. 1, 1½d. per copy, or 1s. per dozen.

**THE ADVANTAGES AND PRIVILEGES OF A TRAINED DISTRICT NURSE, AND THE DUTIES OF THE DISTRICT TOWARDS HER.**

By HENRY C. BURDETT.

No. 2, Same Series, 1½d. per copy, or 1s. per dozen.

**A GREAT MOVEMENT—THE NURSES' CO-OPERATION.**

By Mrs. FURLEY SMITH.

London: The Scientfic Press (Limited), 428, Strand, W.C.

Printed by W. H. and L. Collingridge, City Press, 148 and 149, Aldersgate Street, London, E.C.; and Published by the Proprietors, The Scientific Press (Limited), at 428, Strand, London, W.C.

